# A modality‐specific dysfunction of pain processing in schizophrenia

**DOI:** 10.1002/hbm.24906

**Published:** 2019-12-23

**Authors:** Lili Zhou, Yanzhi Bi, Meng Liang, Yazhuo Kong, Yiheng Tu, Xiangyang Zhang, Yanying Song, Xia Du, Shuping Tan, Li Hu

**Affiliations:** ^1^ CAS Key Laboratory of Mental Health Institute of Psychology, Chinese Academy of Sciences Beijing China; ^2^ Department of Psychology University of Chinese Academy of Sciences Beijing China; ^3^ School of Medical Imaging and Tianjin Key Laboratory of Functional Imaging Tianjin Medical University Tianjin China; ^4^ CAS Key Laboratory of Behavioural Science Institute of Psychology, Chinese Academy of Sciences Beijing China; ^5^ Department of Psychiatry Massachusetts General Hospital and Harvard Medical School Charlestown Massachusetts; ^6^ Psychiatry Research Centre Beijing Huilonguan Hospital Beijing China; ^7^ Department of Pain Management The State Key Clinical Specialty in Pain Medicine, The Second Affiliated Hospital of Guangzhou Medical University Guangzhou China

**Keywords:** electroencephalogram, functional magnetic resonance imaging, pain sensitivity, schizophrenia, sensory processing

## Abstract

Clinical observations showed that schizophrenia (SCZ) patients reported little or no pain under various conditions that are commonly associated with intense painful sensations, leading to a higher risk of morbidity and mortality. However, this phenomenon has received little attention and its underlying neural mechanisms remain unclear. Here, we conducted two experiments combining psychophysics, electroencephalography (EEG), and functional magnetic resonance imaging (fMRI) techniques to investigate neural mechanisms of pain insensitivity in SCZ patients. Specifically, we adopted a stimulus–response paradigm with brief stimuli of different sensory modalities (i.e., nociceptive, non‐nociceptive somatosensory, and auditory) to test whether pain insensitivity in SCZ patients is supra‐modal or modality‐specific, and used EEG and fMRI techniques to clarify its neural mechanisms. We observed that perceived intensities to nociceptive stimuli were significantly smaller in SCZ patients than healthy controls, whereas perceived intensities to non‐nociceptive somatosensory and auditory stimuli were not significantly different. The behavioral results were confirmed by stimulus‐evoked brain responses sampled by EEG and fMRI techniques, thus verifying the modality‐specific nature of the modulation of nociceptive information processing in SCZ patients. Additionally, significant group differences were observed in the spectral power of alpha oscillations in prestimulus EEG and the seed‐based functional connectivity in resting‐state fMRI (seeds: the thalamus and periaqueductal gray that are key nodes in ascending and descending pain pathways respectively), suggesting a possible contribution of cortical–subcortical dysfunction to the phenomenon. Overall, our study provides insight into the neural mechanisms of pain insensitivity in SCZ and highlights a need for systematic assessments of their pain‐related diseases.

## 1 INTRODUCTION

Schizophrenia (SCZ) is a psychiatric disorder marked by a large spectrum of positive symptoms (e.g., hallucination, delusion), negative symptoms (e.g., anhedonia, apathy), and cognitive impairments (Kendler, 2016; Tandon et al., 2013). Despite mental suffering, SCZ patients have an extremely high risk of various pain‐related diseases, such as irritable bowel syndrome, cardiovascular diseases, stroke, fractures, and diabetes mellitus (Fan, Wu, Shen, Ji, & Zhan, 2013; Garakani et al., 2003; Li, Fan, Tang, & Cheng, 2014; Stubbs, Gaughran, et al., 2015; Stubbs, Vancampfort, De Hert, & Mitchell, 2015). However, a lower prevalence of perceived pain is reported in SCZ patients as compared to patients with other psychosis or general population (Chaturvedi, 1987; Engels et al., 2014; Stubbs, Eggermont, et al., 2015; Stubbs, Vancampfort, et al., 2015). This observation could be due to the abnormal pain sensitivity in SCZ patients, which is pervasive but an ignored topic in clinic. The abnormal pain response profile could result in lower possibility for SCZ patients to seek medical help under various conditions normally associated with severe pain (Engels et al., 2014), which could lead to higher morbidity and mortality rates (De Hert, Cohen, et al., 2011; De Hert, Correll, et al., 2011; Dworkin, 1994; Jarcho, Mayer, Jiang, Feier, & London, 2012). These facts prompt the urgency to explore the interrelationship between pain and SCZ.

Although there are a few conflicting results (Bonnot, Anderson, Cohen, Willer, & Tordjman, 2009; Girard, Plansont, Bonnabau, & Malauzat, 2011; Guieu, Samuelian, & Coulouvrat, 1994), the majority of previous studies has demonstrated that pain sensitivity is decreased in SCZ patients, mainly supported by three lines of evidence. First, clinical case reports have shown that SCZ patients have little pain complaints in conditions that are commonly associated with intense painful sensations (e.g., bacterial peritonitis caused by perforated appendix) (Apter, 1981; Murakami et al., 2010; Murthy, Narayan, & Nayagam, 2004; Potvin, Stip, & Marchand, 2016; Rosenthal, Porter, & Coffey, 1990; Virit, Savas, & Altindag, 2008). Second, population studies have demonstrated a remarkably high prevalence of reduced or absent pain symptoms in SCZ patients (Singh, Giles, & Nasrallah, 2006; Torrey, 1979), but an extremely low prevalence of SCZ diagnosis in chronic pain patients (Fishbain, Goldberg, Meagher, Steele, & Rosomoff, 1986; Reich, Tupin, & Abramowitz, 1983). Third, empirical studies have described increased pain threshold or tolerance in SCZ patients during various types of nociceptive stimulations (e.g., heat, cold, electrical) (Blumensohn, Ringler, & Eli, 2002; Jochum et al., 2006; Kudoh, Ishihara, & Matsuki, 2000). Therefore, it has been suggested that pain insensitivity could be considered as a potential endophenotype of SCZ (Stubbs, Thompson, et al., 2015).

Notably, a few pilot studies directly compared the neural activity evoked by nociceptive stimuli between patients and healthy controls (HC), and revealed abnormal pain processing at cortical level during a psychotic state in SCZ (de la Fuente‐Sandoval, Favila, Gomez‐Martin, Leon‐Ortiz, & Graff‐Guerrero, 2012; de la Fuente‐Sandoval, Favila, Gomez‐Martin, Pellicer, & Graff‐Guerrero, 2010; Linnman, Coombs 3rd, Goff, & Holt, 2013). These findings suggest that abnormal pain sensitivity in SCZ may be consequent upon a general deficit of sensory information processing (Javitt & Freedman, 2015), which has been frequently reported in multiple sensory modalities (e.g., auditory, somatosensory, visual) (Braff, Light, & Swerdlow, 2007; Butler et al., 2007; Levy et al., 2000; Turetsky et al., 2008). However, competing evidence has shown the intact function of sensory detection in some modalities (e.g., auditory detection thresholds or visual acuity) in SCZ (Carter et al., 2017; Javitt & Freedman, 2015), suggesting an alternative hypothesis that mechanisms underlying pain insensitivity in SCZ may not be generic across sensory modalities, but rather specific to nociception.

To test whether pain insensitivity in SCZ is a supra‐modal or modality‐specific phenomenon, we conducted two experiments combining with psychophysics, electroencephalography (EEG), and functional magnetic resonance imaging (fMRI) techniques. In Experiment 1 (*n* = 42), we used a stimulus–response paradigm to obtain stimulus‐evoked and prestimulus EEG data and compared sensory processing across three different modalities (i.e., nociceptive, non‐nociceptive somatosensory, and auditory) between SCZ and HC. In Experiment 2 (*n* = 40), we further compared fMRI blood‐oxygen‐level dependent (BOLD) responses to nociceptive stimuli and seed‐based resting‐state functional connectivity (RSFC) between SCZ and HC.

## 2 METHODS

### 2.1 Experiment 1 (EEG)

#### 2.1.1 Subjects

Twenty‐one right‐handed patients, diagnosed with SCZ according to DSM‐V (Bhati, 2013) at the inpatient and outpatient psychiatric services of Beijing Huilonguan Hospital, were recruited through clinical assessments in Experiment 1. Further inclusion criteria were as follows: illness duration longer than 2 years, considered clinically stable by their treating physician, no electric compulsive treatment in the past 6 months. Exclusion criteria included concomitant severe medical or neurological illness, comorbidity of any other DSM‐V Axis I disorder, past or current alcohol abuse, high suicidal risk, and contraindications of MRI scanning. Twenty‐one age‐gender matched, right‐handed HC without positive personal/family history of psychosis or current concomitant physical pain were recruited from the local communities. Other exclusion criteria for HC were identical to SCZ. All subjects provided written informed consent. The experiment was approved by the Ethics Review Board at the Beijing Huilonguan Hospital and registered with ChiCTR‐BOC‐17013972 in the Chinese Clinical Trial Registry. All experimental procedures were carried out in accordance with the Declaration of Helsinki.

#### 2.1.2 Clinical assessments

For SCZ, clinical assessments included age at illness onset, duration of illness, personal and family psychopathology, substance use, and medication. The personal and family psychopathology was assessed using the Positive and Negative Syndrome Scale (PANSS) (Kay, Fiszbein, & Opler, 1987). Substance use, particularly the duration and daily dose of smoke, was evaluated, as nicotine can modify subjective pain sensitivity (Girdler et al., 2005). Type and dosage of antipsychotic were recorded, and the daily antipsychotic dose was converted into chlorpromazine equivalents (Gardner, Murphy, O'Donnell, Centorrino, & Baldessarini, 2010). Clinical assessments on HC, which included personal and family psychopathology and substance use, were conducted using a semi‐structural clinical interview. Sociodemographic and clinical data are summarized in Table 1.

**Table 1:** The characteristics of subjects in Experiments 1 and 2

		Experiment 1		Experiment 2	
Variables	Categories	SCZ (*n* = 21)	HC (*n* = 21)	SCZ (n = 20)	HC (n = 20)
Age, y		37.6 ± 7.9	34.4 ± 7.2	37.9 ± 7.1	34.4 ± 6.5
Education, y		13.7 ± 2.5	14.6 ± 3.5	14.0 ± 2.6	14.3 ± 3.6
Gender, no. (%)	Male	16 (76.2%)	16 (76.2%)	15 (75.0%)	16 (80.0%)
	Female	5 (23.8%)	5 (23.8%)	5 (25.0%)	4 (20.0%)
Ethnicity, no. (%)	Han	18 (85.7%)	21 (100%)	18 (90.0%)	20 (100%)
	Minority	3 (14.3%)	0 (0%)	2 (10.0%)	0 (0%)
Onset‐age of SCZ, y		22.0 ± 6.8		21.9 ± 6.9	
Duration of SCZ, y		15.9 ± 8.0		16.6 ± 7.8	
Positive symptoms		13.2 ± 5.2		13.3 ± 5.1	
Negative symptoms		16.4 ± 7.5		16.1 ± 7.6	
General psychosis		25.7 ± 5.9		24.9 ± 5.2	
PANSS		55.3 ± 12.9		54.3 ± 13.1	
Personal/family history of psychosis, no. (%)		0 (0%)	0 (0%)	0 (0%)	0 (0%)
Duration of smoke, y		11.1 ± 6.7 (*n* = 9)	14.4 ± 9.9 (*n* = 7)	9.3 ± 5.4 (*n* = 10)	14.4 ± 9.9 (*n* = 7)
Daily dose of smoke, no.		8.7 ± 7.6	10.0 ± 9.5	8.3 ± 7.3	10.0 ± 9.5
Antipsychotic dosage,^a^ mg		608.5 ± 262.9		659.5 ± 269.7	
First‐generation antipsychotic,^b^ no. (%)		2 (9.5%)		2 (10.0%)	
Second‐generation antipsychotic,^b^ no. (%)		19(90.5%)		18(90.0%)	

*Note*: Data are expressed in mean ± SD.

a Antipsychotic dosage has been converted into chlorpromazine equivalents.

b Some patients were taking both first‐generation and second‐generation antipsychotics.

#### 2.1.3 Sensory stimuli

*Nociceptive stimuli* were pulses of radian heat generated by an infrared neodymium yttrium aluminum perovskite laser (Deka: Stimul 1,340; wavelength: 1.34 μm; pulse duration: 4 ms; Electronic Engineering, Italy). At this wavelength and pulse duration, laser stimuli activate directly nociceptive terminals in the most superficial skin layers in a synchronized fashion (Iannetti, Zambreanu, & Tracey, 2006). A He–Ne laser pointed to the area to be stimulated. The laser beam was set at a diameter of ~7 mm by focusing lenses connected to the optic fiber, with a fixed stimulus intensity of 3.5 J to elicit a painful pinprick sensation (Bromm & Treede, 1984). To prevent fatigue or sensitization of the nociceptors, the laser beam target was manually shifted by at least 1 cm in a random direction after each stimulus. Laser pulses were delivered to a squared area (4 × 4 cm^2^) on the dorsum of left hand. *Non‐nociceptive somatosensory stimuli* were constant current square‐wave electrical pulses generated by an electrical stimulator (pulse duration: 1 ms; SXC‐4A, Sanxia Technique Inc., China). The electrical pulses were delivered via a pair of surface round electrodes (diameter: 16 mm; inter‐electrode distance: 1 cm) placed over the median nerve at the left wrist. The stimulus intensity was fixed at 7 mA to elicit a nonpainful tactile sensation. *Auditory stimuli* were brief 1,000‐Hz pure tones (~70 dB; duration: 50 ms; 5‐ms rise and fall time) delivered binaurally through custom‐built headphones (Sennheiser, HD201, Germany).

#### 2.1.4 EEG experimental design

**Figure 1:**
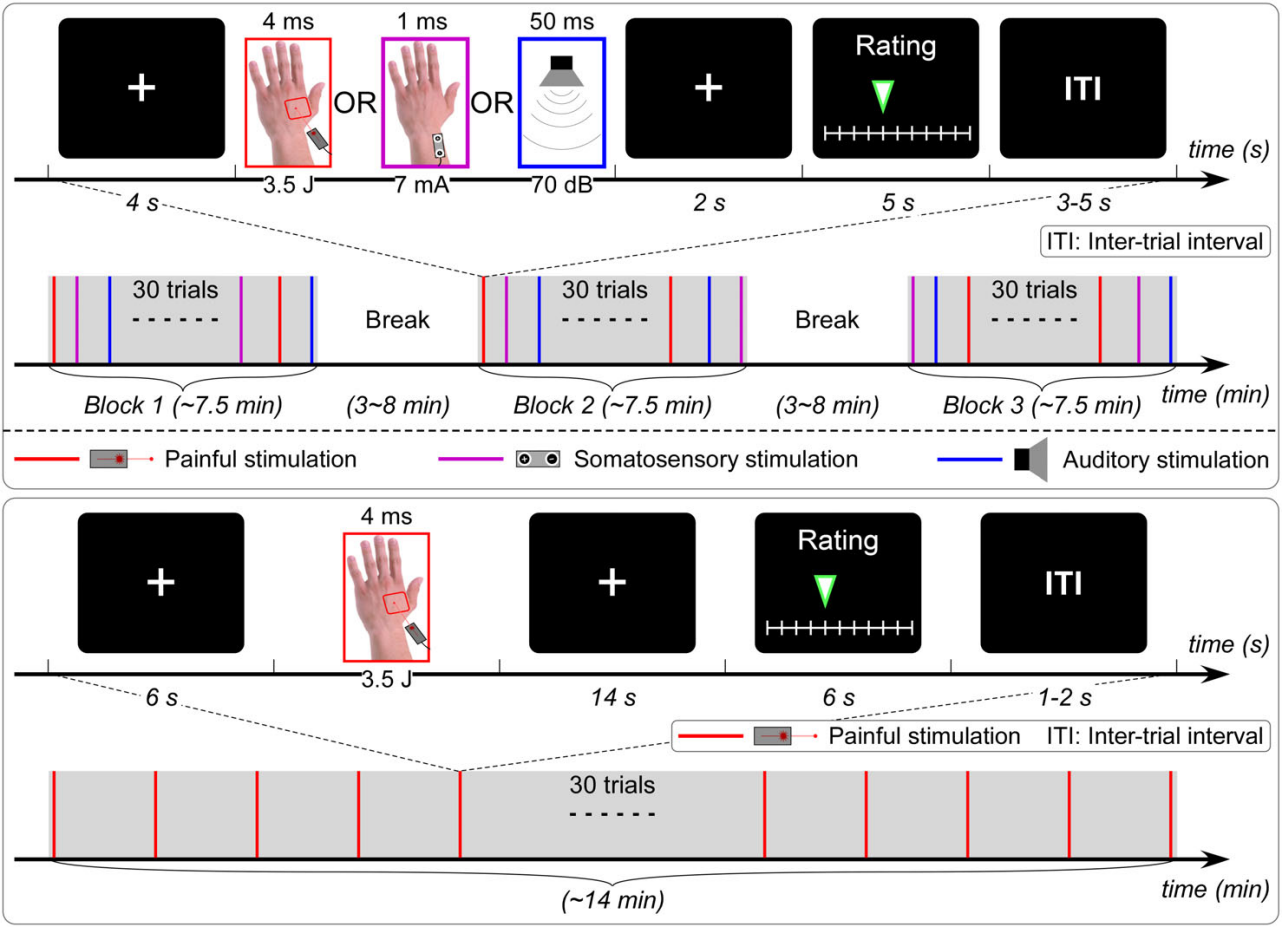
EEG and fMRI experimental paradigms. *Top panel*: EEG experiment (Experiment 1) was composed of three blocks, and in each block 30 trials with transient stimuli belonging to three different sensory modalities (i.e., nociceptive, non‐nociceptive somatosensory, and auditory) were delivered in a pseudorandom order. Subjects were allowed to have a short break (3–8 min) after each block. Each trial started with a 4‐s fixation of white cross‐centered on the screen, and followed by the delivery of a sensory stimulus. A visual cue presented 2 s after the sensory stimulus prompted the subjects to verbally rate the perceived intensity within 5 s on a 11‐point NRS ranging from 0 (no sensation) to 10 (unbearable sensation). The inter‐trial interval (ITI) was 3–5 s. *Bottom panel*: fMRI experiment (Experiment 2) contained a single block of 30 trials with transient nociceptive stimuli. Each trial started with a 6‐s fixation of the white cross‐centered on the screen, and followed by the delivery of a nociceptive stimulus. A visual cue presented 14 s after the nociceptive stimulus prompted the subjects to rate the perceived intensity within 6 s on the same 11‐point NRS. The ITI was 1–2 s

The EEG experiment consisted of three blocks (Figure 1, top panel). In each block, 30 sensory stimuli, belonging to three different sensory modalities (i.e., nociceptive, non‐nociceptive somatosensory, and auditory), were delivered in a pseudorandom order. For each sensory modality, we delivered 10 stimuli in each block (30 stimuli per sensory modality in total). A typical trial started with a 4‐s fixation of white cross‐centered on the screen, which was followed by the delivery of a sensory stimulus. A visual cue presented 2 s after the sensory stimulus prompted the subjects to verbally rate the perceived intensity within 5 s on an 11‐point Numeric Rating Scale (NRS) ranging from 0 to 10. For nociceptive stimuli, “0” represented “not painful at all” and “10” represented “extremely painful”; for non‐nociceptive somatosensory stimuli, “0” represented “no sensation at all” and “10” represented “extremely strong/intense sensation”; for auditory stimuli, “0” represented “no sound at all” and “10” represented “extremely loud”. The inter‐trial interval (ITI) was 3–5 s. Subjects could have a short break (3–8 min) after each block. During the experiment, all subjects were required to keep themselves awake and eyes‐open, and focused themselves on detecting the perceived intensity for each sensory stimulus.

#### 2.1.5 EEG data acquisition

Subjects were seated in a comfortable chair in a silent room, and were instructed to focus on the stimuli, keep their eyes open, and gaze at a fixation point on the screen. EEG data were recorded using 64 Ag‐AgCl scalp electrodes placed according to the International 10–20 system (ANT Neuro; pass band: 0.01–100 Hz; sampling rate: 1,000 Hz). The nose was used as the reference, and electrode impedances were kept lower than 10 kΩ. To monitor eyeblinks and ocular movements, electrooculographic (EOG) signals were simultaneously recorded from two surface electrodes, one placed over the left lower eyelid and the other placed lateral to the outer canthus of the left eye.

#### 2.1.6 EEG data preprocessing

EEG data were preprocessed using EEGLAB, an open source toolbox running in the MATLAB environment. Continuous EEG data were band‐pass filtered between 1 and 30 Hz, and segmented into epochs using a time window of 1,500 ms, ranging from 500 ms prestimulus to 1,000 ms poststimulus. Baseline correction was performed using the prestimulus interval. Trials contaminated by eye‐blinks and movements were corrected using an independent component analysis (ICA) algorithm (Delorme & Makeig, 2004).

#### 2.1.7 Event‐related potentials: Time‐domain analysis

For each subject, epochs belonging to the same sensory modality were averaged, yielding three average waveforms time‐locked to the stimulus onset. For laser‐evoked potentials (LEPs), peak latencies and amplitudes of N1, N2, and P2 waves were measured from the average waveform. N2 and P2 waves were defined as the most negative and positive deflections between 150 and 500 ms after stimulus onset at the central electrode (Cz‐nose, vertex potentials), respectively (Kunde & Treede, 1993; Valentini et al., 2012). N1 wave, defined as the most negative deflection preceding the N2 wave, can be optimally detected at the central electrode contralateral to the stimulated side referenced to Fz (C4‐Fz) (Hu, Mouraux, Hu, & Iannetti, 2010; Valentini et al., 2012). For non‐nociceptive somatosensory‐evoked potentials (SEPs) and auditory‐evoked potentials (AEPs), peak latencies and amplitudes of N2 and P2 waves were measured from the average waveforms. For both SEPs and AEPs, N2 and P2 waves were defined as the most negative and positive deflections between 100 and 400 ms after stimulus onset at the central electrode (Cz‐nose, vertex potentials), respectively (Mouraux & Iannetti, 2009; Peng, Hu, Zhang, & Hu, 2012). Please note that vertex potentials elicited by intense stimuli belonging to non‐nociceptive somatosensory and auditory modalities are functionally similar to the N2–P2 complex in LEP responses (Mouraux & Iannetti, 2009). For this reason, we used the same nomenclatures (i.e., N2 and P2 waves) for all sensory modalities in the present study. Single‐subject average waveforms of each sensory modality were averaged across subjects to obtain group‐level waveforms. Group‐level scalp topographies at the peak latency of all waves were computed by spline interpolation.

### 2.2 Experiment 2 (fMRI)

#### 2.2.1 Subjects

Twenty patients and 20 age‐gender matched HC were recruited in Experiment 2, and most of them (15 patients and 17 HC) also participated in Experiment 1. The inclusion/exclusion criteria and other experimental requirements were identical to Experiment 1. Sociodemographic and clinical data are summarized in Table 1.

#### 2.2.2 fMRI experimental design

The fMRI experiment consisted of a resting‐state fMRI session and a task fMRI session (Figure 1, bottom panel). For the resting‐state fMRI scanning session, subjects were required to lay supine in the scanner, and kept their eyes fixed on a white cross centered on the screen for ~10 min. For the task fMRI scanning session, the paradigm was similar to that in Experiment 1, but only nociceptive stimuli were delivered as the main aim of this experiment was to explore the neural mechanisms underlying the alterations of pain sensitivity in SCZ. Thirty nociceptive stimuli (laser pulses were also generated by Stimul 1,340, which is an MRI‐compatible device) were delivered to a squared area (4 × 4 cm^2^) on the dorsum of the left hand. Other laser parameters (i.e., wavelength, pulse duration, beam diameter, and stimulus intensity) were identical to Experiment 1. Please note that, the laser beam target was manually shifted by an experimenter in the scanning room for at least 1 cm in a random direction after each stimulus to prevent fatigue or sensitization of the nociceptors. Each trial started with a 6‐s fixation of the white cross‐centered on the screen, followed by the delivery of a nociceptive stimulus. Fourteen seconds after the delivery of nociceptive stimulus, a visual cue was presented to prompt the subjects to rate the perceived intensity within 6 s on the same 11‐point NRS by pressing buttons on a shank in their right hand. The ITI was 1–2 s.

#### 2.2.3 MRI data acquisition

Both structural and functional MRI data were acquired on a 3.0 Tesla Siemens Prisma magnetic resonance scanner (Erlangen, Germany) with a standard 64‐ch head coil at Beijing HuiLongGuan Hospital. For each subject, a T1‐weighted structural image (echo time = 3.97 ms, repetition time = 1,900 s, voxel size = 1 × 1 × 1 mm^3^, in‐plane matrix size = 240 × 240, slices = 192, field of view = 192 × 192 mm^2^) was acquired to exclude the possibility of clinically silent lesions for all subjects and for use of spatial registration during the functional imaging data analyses. A whole‐brain gradient‐echo, echo‐planar imaging (GE‐EPI) sequence was used for obtaining functional data (echo time = 30 ms, repetition time = 2,000 ms, flip angle = 90°, field of view = 224 × 224 mm^2^, matrix = 64 × 64, 33 contiguous slices with thickness of 3.5 mm) of 300 volumes for resting‐state fMRI session and 414 volumes for task fMRI session.

#### 2.2.4 fMRI data preprocessing

fMRI data were preprocessed using FSL tools (FMRIB's Software Library, Version 6.00, http://www.fmrib.ox.ac.uk/fsl). The same preprocessing procedures were applied to the resting‐state and task fMRI data, which included motion correction (mcflirt), distortion correction with field map (FUGUE) (Jenkinson, Bannister, Brady, & Smith, 2002), nonbrain tissue removal using Brain Extraction Tool (BET), spatial smoothing with a Gaussian kernel of 5‐mm full‐width at half‐maximum (FWHM), high‐pass temporal filtering using a Gaussian‐weighted least‐squares straight line fitting with sigma of 100 s. For the resting‐state fMRI data, ICA‐based denoising was performed (Beckmann & Smith, 2004) for each subject to remove the artifacts, including head motion, white matter, cerebrospinal fluid, high‐frequency noise, slice dropouts, gradient instability, EPI ghosting, and field inhomogeneities, etc.

#### 2.2.5 Task fMRI: General linear model analysis

Each single‐subject task fMRI data were modeled on a voxel‐by‐voxel basis through a general linear model (GLM) approach (Woolrich, Ripley, Brady, & Smith, 2001). The fMRI time series were modeled using a series of regressors including the events of interest (i.e., occurrence of nociceptive stimuli) convolved with a gamma hemodynamic response function, its temporal derivative, and six head motion parameters estimated during motion correction. For each subject, the contrast corresponding to the regressor of nociceptive stimuli was used to assess the BOLD responses associated with nociceptive stimuli. As all contrasts were constructed in individual functional space, a two‐stage spatial registration procedure was applied to normalize individual functional images to the Montreal Neurological Institute (MNI) standard space: single‐subject low‐resolution functional images were first co‐registered to their corresponding high‐resolution structural images using FMRIB's Linear Image Registration Tool (FLIRT) (Jenkinson et al., 2002) and then transformed to a standard brain (MNI 152 2 mm template) using FLIRT and FMRIB's Non‐linear Image Registration Tool (FNIRT) (Andersson, Jenkinson, & Smith, 2007). Group‐level statistical analyses were carried out using a mixed‐effect approach (FLAME, FMRIB's Local Analysis of Mixed Effects) (Beckmann, Jenkinson, & Smith, 2003; Woolrich, Behrens, Beckmann, Jenkinson, & Smith, 2004). The single‐subject contrast maps entered a one‐sample *t* test to obtain the group‐level brain responses to nociceptive stimuli for each group (SCZ and HC). The differences between SCZ and HC were assessed using the independent‐sample *t* test of single‐subject contrast maps. The statistical images were thresholded using cluster‐forming correction determined by *Z* > 2.3 and a corrected cluster significance threshold of *p* < .05 (Worsley, 2003).

#### 2.2.6 Resting‐state fMRI: Functional connectivity analysis

Given that the thalamus and periaqueductal gray (PAG) are key nodes in the ascending and descending pain modulation pathways respectively (Ab Aziz & Ahmad, 2006; Basbaum & Fields, 1984), and showed significant differences in BOLD responses to nociceptive stimuli between SCZ and HC detected in the task fMRI GLM analysis, these two brain regions were defined as two seed ROIs for the functional connectivity analysis of resting‐state fMRI data. The ROI of thalamus was defined from the Harvard Oxford subcortical structural atlas (Frazier et al., 2005), which are population‐based probability atlas in MNI 152 standard space. The ROI of PAG was defined from Duvernoy's atlas of the Human Brainstem and Cerebellum (Naidich et al., 2009) in MNI 152 standard space (Ezra, Faull, Jbabdi, & Pattinson, 2015). To investigate the RSFC of each ROI in individual functional space using FEAT (FMRI Expert Analysis Tool, Version 6.00), the two seed ROIs in standard space were first transformed into individual low‐resolution functional space via inverted registration files with nearest‐neighbor interpolation. Voxel‐wise seed‐based RSFC analyses were completed using standard methods (Segerdahl, Themistocleous, Fido, Bennett, & Tracey, 2018) for each seed ROI as follows. The mean time series of a given ROI was set as a connectivity EV with realignment parameters, averaged white matter signal and cerebrospinal fluid signal as the nuisance regressors. Functional connectivity maps were transformed straight into standard space following the same registration steps as task fMRI. The differences in the RSFC of each ROI between SCZ and HC were assessed using the independent‐sample *t* test. The statistical images were thresholded using cluster‐forming correction determined by *Z* > 2.3 and a corrected cluster significance threshold of *p* < .05 (Worsley, 2003).

#### 2.2.7 Statistics

The average ratings of the perceived intensity and event‐related EEG responses were compared using two‐way mixed‐design analyses of variance (ANOVA), with “modality” (three levels: nociceptive, non‐nociceptive somatosensory, and auditory) as a within‐subject factor and “group” (two levels: SCZ and HC) as a between‐subject factor. The statistical P values were adjusted with Greenhouse–Geisser correction to avoid violation of the sphericity assumption, when necessary. When the main effects or the interaction reach statistical significance, post hoc pairwise comparisons with Bonferroni correction were performed. The peak latency and amplitude of N1 wave in LEPs were compared between groups using an independent‐sample *t* test.

In addition, Pearson's correlation analyses were performed between the ratings of perceived pain and a series of variables, including: (1) laser‐evoked EEG responses (i.e., N1, N2, and P2 amplitudes), (2) prestimulus EEG oscillations (i.e., lower and higher *α* oscillations, which were detailed in the Supplementary materials), (3) laser‐evoked BOLD responses (BOLD responses in brain regions showed significant group differences), and (4) seed‐based RSFC (RSFC between thalamus/PAG and clusters showed significant group differences). To quantify the relationship between EEG and fMRI measures at resting state, Pearson's correlation analyses were also performed between prestimulus EEG oscillations and seed‐based RSFC.

## 3 RESULTS

### 3.1 Psychophysics

**Figure 2:**
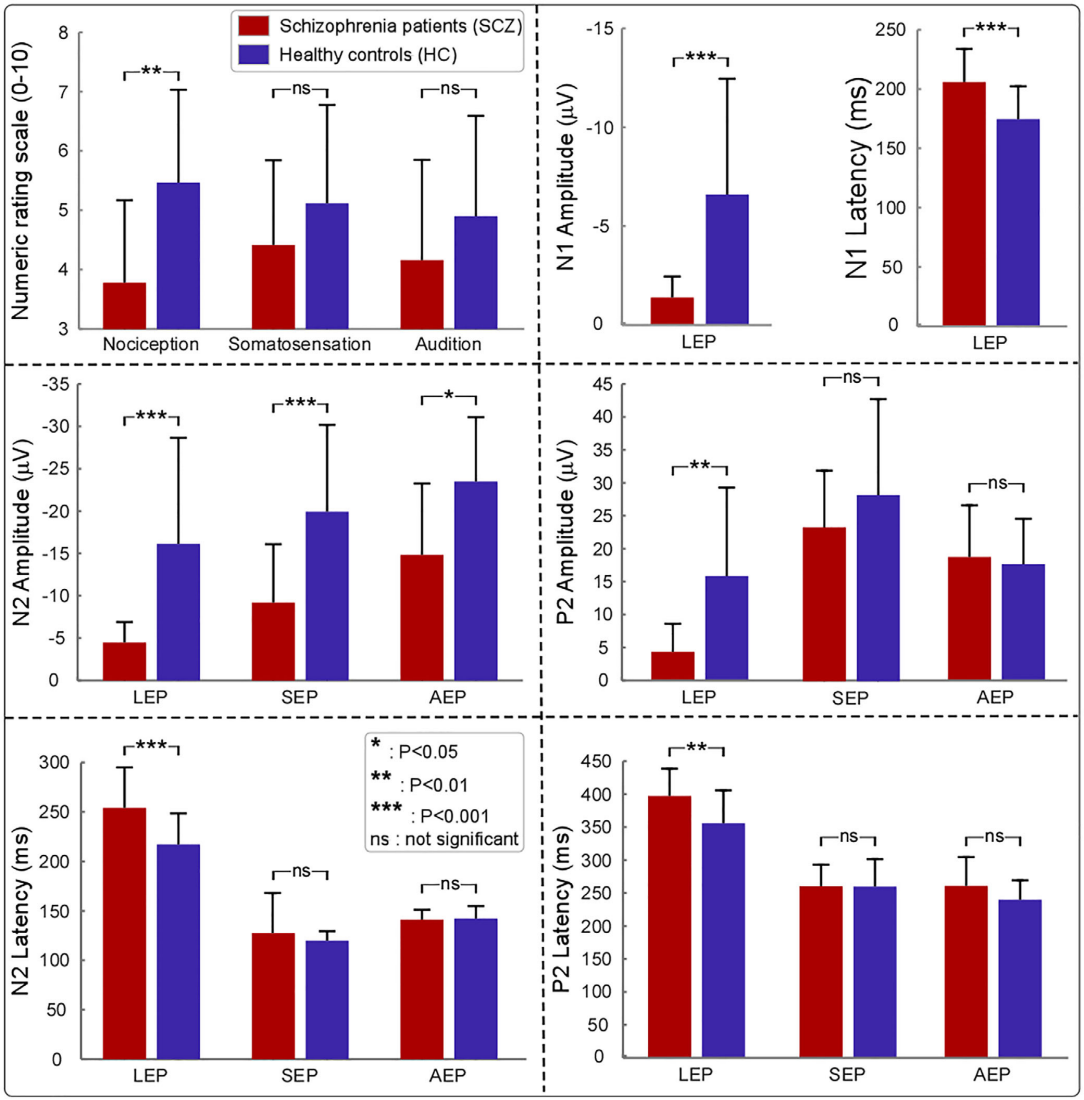
Comparison of behavioral variables and electrophysiological features between SCZ and HC. *Top left panel*: Perceived intensities to different sensory stimuli. While the perceived intensities to nociceptive stimuli were significantly smaller for SCZ compared to HC, the perceived intensities to non‐nociceptive somatosensory and auditory stimuli showed no significant differences between the two groups. *Top right*, *middle*, *and bottom panels*: Event‐related EEG responses to different sensory stimuli in the time domain. Latencies and amplitudes of all LEP waves (i.e., N1, N2, and P2) were significantly different between SCZ and HC. In contrast, no significant differences between SCZ and HC were found in N2 latency, P2 latency, and P2 amplitude of SEPs and AEPs. Notably, for both SEPs and AEPs, N2 amplitudes were significantly smaller in SCZ than HC (*p < .05; **p < .01; ***p < .001; ns: not significant)

For both experiments, no significant differences were observed between SCZ and HC in age, years of education, and substance use (Table 1). For ratings of perceived intensity to sensory stimuli, significant main effect of “group” (*F*_[1,40]_ = 7.002, *p* = .012, $\eta_{p}^{2}$ = 0.149) and interaction between “group” and “modality” (*F*_[2,40]_ = 3.573, *p* = .033, $\eta_{p}^{2}$ = 0.082) were observed. Post hoc pairwise comparisons revealed that the perceived intensities to nociceptive stimuli in SCZ were significantly smaller than that in HC (*p* = .006), while the perceived intensities to non‐nociceptive somatosensory (*p* = 1.000) and auditory (*p* = 1.000) stimuli were not significantly different between the two groups (Table 2; Figure 2, top left panel), indicating a selective reduction of pain sensitivity in SCZ patients.

**Table 2:** Comparisons of psychophysics and electrophysiological features between SCZ and HC

Features	Variables	SCZ (*n* = 21)	HC (*n* = 21)
Perceived intensities	Nociceptive	3.7 ± 1.4	5.4 ± 1.5
	Non‐nociceptive somatosensory	4.4 ± 1.4	5.1 ± 1.6
	Auditory	4.2 ± 1.6	4.8 ± 1.6
LEP responses	N1 latency (ms)	206.0 ± 27.9	174.6 ± 27.7
	N1 amplitude (μV)	−1.4 ± 1.1	−6.6 ± 5.9
	N2 latency (ms)	254.1 ± 41.0	217.1 ± 31.5
	N2 amplitude (μV)	−4.5 ± 2.4	−16.1 ± 12.5
	P2 latency (ms)	397.3 ± 41.3	356.0 ± 49.7
	P2 amplitude (μV)	4.3 ± 4.3	15.9 ± 13.4
SEP responses	N2 latency (ms)	127.4 ± 40.6	119.9 ± 9.6
	N2 amplitude (μV)	−9.2 ± 6.9	−19.9 ± 10.3
	P2 latency (ms)	260.3 ± 32.9	256.0 ± 41.7
	P2 amplitude (μV)	23.4 ± 8.6	28.3 ± 14.6
AEP responses	N2 latency (ms)	141.3 ± 9.9	142.4 ± 12.5
	N2 amplitude (μV)	−14.8 ± 8.4	−23.5 ± 7.6
	P2 latency (ms)	260.9 ± 43.8	240.0 ± 29.3
	P2 amplitude (μV)	18.7 ± 7.9	17.6 ± 6.9

*Note*: Data are expressed in mean ± SD.

### 3.2 Event‐related EEG responses to sensory stimuli in the time domain

**Figure 3:**
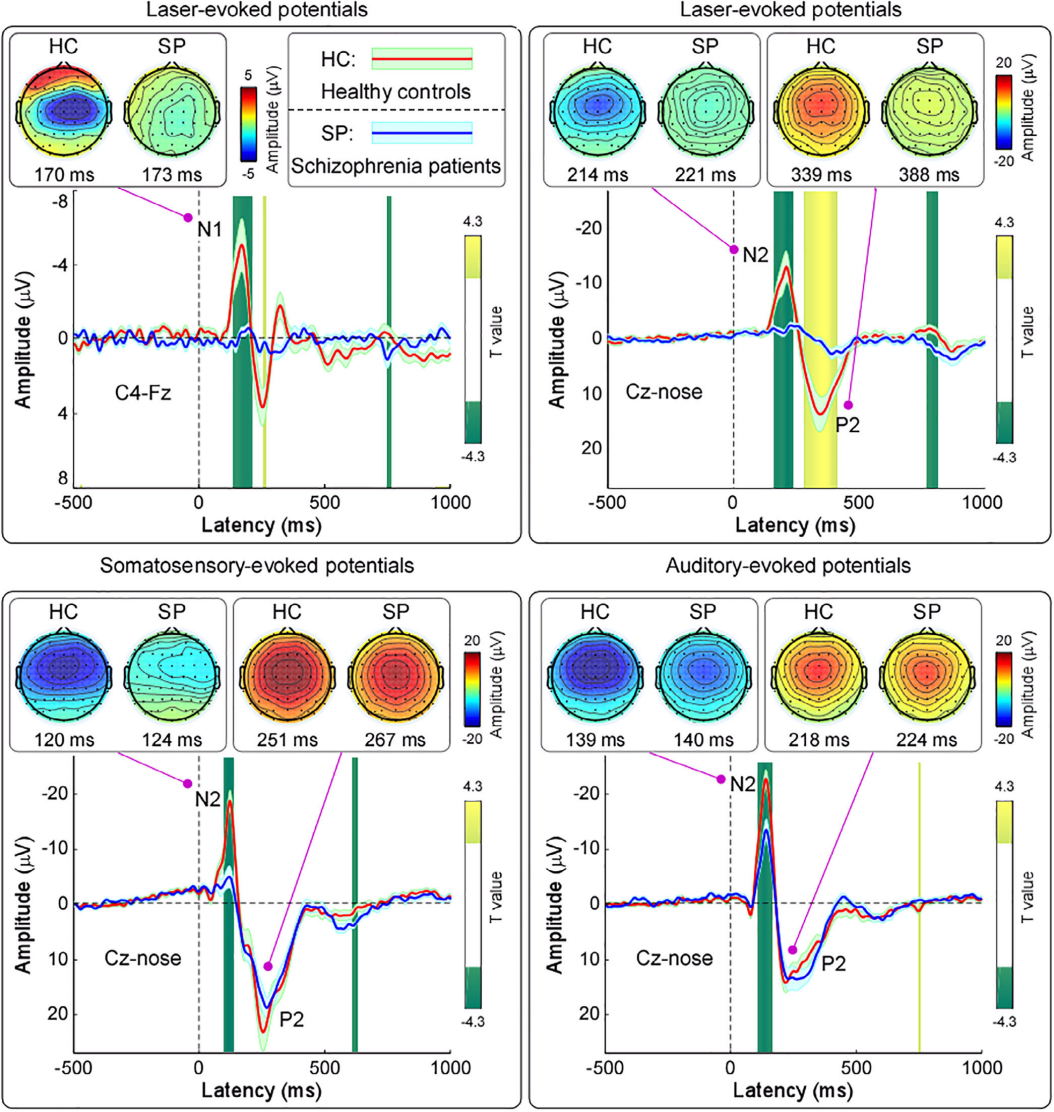
Group‐level event‐related EEG responses to sensory stimuli in the time domain. *Top panels*: Group‐level LEP waveforms and scalp topographies of N1 wave (C4‐Fz) and N2‐P2 complex (Cz‐nose). Data from SCZ and HC are displayed in blue and red, respectively. Time intervals with significant difference between the two groups are marked in green and yellow for negative and positive *T* values, respectively. Scalp topographies are plotted at the peak latency of each wave. *Bottom left panel*: Group‐level SEP waveforms and scalp topographies of N2 and P2 waves (Cz‐nose). *Bottom right panel*: Group‐level AEP waveforms and scalp topographies of N2 and P2 waves (Cz‐nose)

Group‐level LEP waveforms and scalp topographies of N1, N2, and P2 waves in the time domain are shown in the top panel of Figure 3. In line with previous studies (Hu, Cai, Xiao, Luo, & Iannetti, 2014; Valentini et al., 2012), scalp topographies of the N1 wave were maximal at central electrodes contralateral to the stimulated hand, as it has been demonstrated that N1 wave is generated in the contralateral primary somatosensory/motor cortices. Scalp topographies of the N2 wave were maximal at the vertex and extended bilaterally towards temporal regions, and scalp topographies of the P2 wave were more centrally distributed (Mouraux & Iannetti, 2009). Group‐level SEP waveforms and scalp topographies of N2 and P2 waves in the time domain, which were maximal at the vertex, are shown in the bottom left panel of Figure 3. Group‐level AEP waveforms and scalp topographies of N2 and P2 waves in the time domain, which were maximal at the vertex, are shown in the bottom right panel of Figure 3.

For N2 amplitude, significant main effect of “group” (*F*_[1,40]_ = 23.194, *p* < .001, $\eta_{p}^{2}$ = 0.367) and “modality” (*F*_[2,40]_ = 21.512, *p* < .001, $\eta_{p}^{2}$ = 0.350) were observed. Specifically, N2 amplitude was significantly smaller in SCZ than in HC regardless of sensory modality (LEP: *p* < .001, SEP: *p* = .001, AEP: *p* = .021; Table 2; Figure 2, middle left panel). There was no significant interaction between "group" and "modality" on N2 amplitude (*F*_[2,40]_ = 1.005, *p* = .322, $\eta_{p}^{2}$ = 0.025). For N2 latency, significant main effects of “group” (*F*_[1,40]_ = 7.140, *p* = .011, $\eta_{p}^{2}$ = 0.151) and “modality” (*F*_[2,40]_ = 215.795, *p* < .001, $\eta_{p}^{2}$ = 0.844), as well as the interaction between “group” and “modality” (*F*_[2,40]_ = 5.955, *p* = .007, $\eta_{p}^{2}$ = 0.130) were observed. Post hoc pairwise comparisons showed that significant group difference of N2 latency was only observed in LEPs (SCZ > HC, *p* = .001; Table 2; Figure 2, bottom left panel).

For P2 amplitude, significant main effects of “group” (*F*_[1,40]_ = 4.096, *p* = .050, $\eta_{p}^{2}$ = 0.093) and “modality” (*F*_[2,40]_ = 54.285, *p* < .001, $\eta_{p}^{2}$ = 0.576), as well as the interaction between “group” and “modality” (*F*_[2,40]_ = 18.767, *p* < .001, $\eta_{p}^{2}$ = 0.319) were observed. Post hoc pairwise comparisons showed that significant group difference of P2 amplitude was only observed in LEPs (SCZ < HC, *p* = .004; Table 2; Figure 2, middle right panel). For P2 latency, significant main effects of “group” (*F*_[1,40]_ = 6.142, *p* = .018, $\eta_{p}^{2}$ = 0.133) and “modality” (*F*_[2,40]_ = 155.848, *p* < .001, $\eta_{p}^{2}$ = 0.796), as well as the interaction between “group” and “modality” (*F*_[2,40]_ = 3.313, *p* = .041, $\eta_{p}^{2}$ = 0.076) were observed. Post hoc pairwise comparisons showed that significant group difference of P2 latency was only observed in LEPs (SCZ > HC, *p* = .018; Table 2; Figure 2, bottom right panel).

In LEPs, N1 amplitude was significantly smaller in SCZ than in HC (*t*[21.322] = 4.014, *p* < .001, Cohen's *d* = 1.269); N1 latency was significant larger in SCZ than in HC (*t*[40] = 3.657, *p* = .001, Cohen's *d* = 1.156; Table 2; Figure 2, top right panel).

In summary, all tested variables in the event‐evoked EEG responses to nociceptive stimuli were significantly different between SCZ and HC, represented as smaller peak amplitude and longer peak latency in SCZ than in HC. Whereas N2 latency, P2 latency and amplitude in the event‐evoked EEG responses to non‐nociceptive somatosensory and auditory stimuli were not significantly different between the two groups, N2 amplitude was significantly smaller in SCZ than HC. These results demonstrated that different from non‐nociceptive somatosensory and auditory modalities, there is an overall dysfunction of nociceptive information processing in SCZ, which is in line with the behavioral data.

### 3.3 Event‐evoked BOLD responses

**Figure 4:**
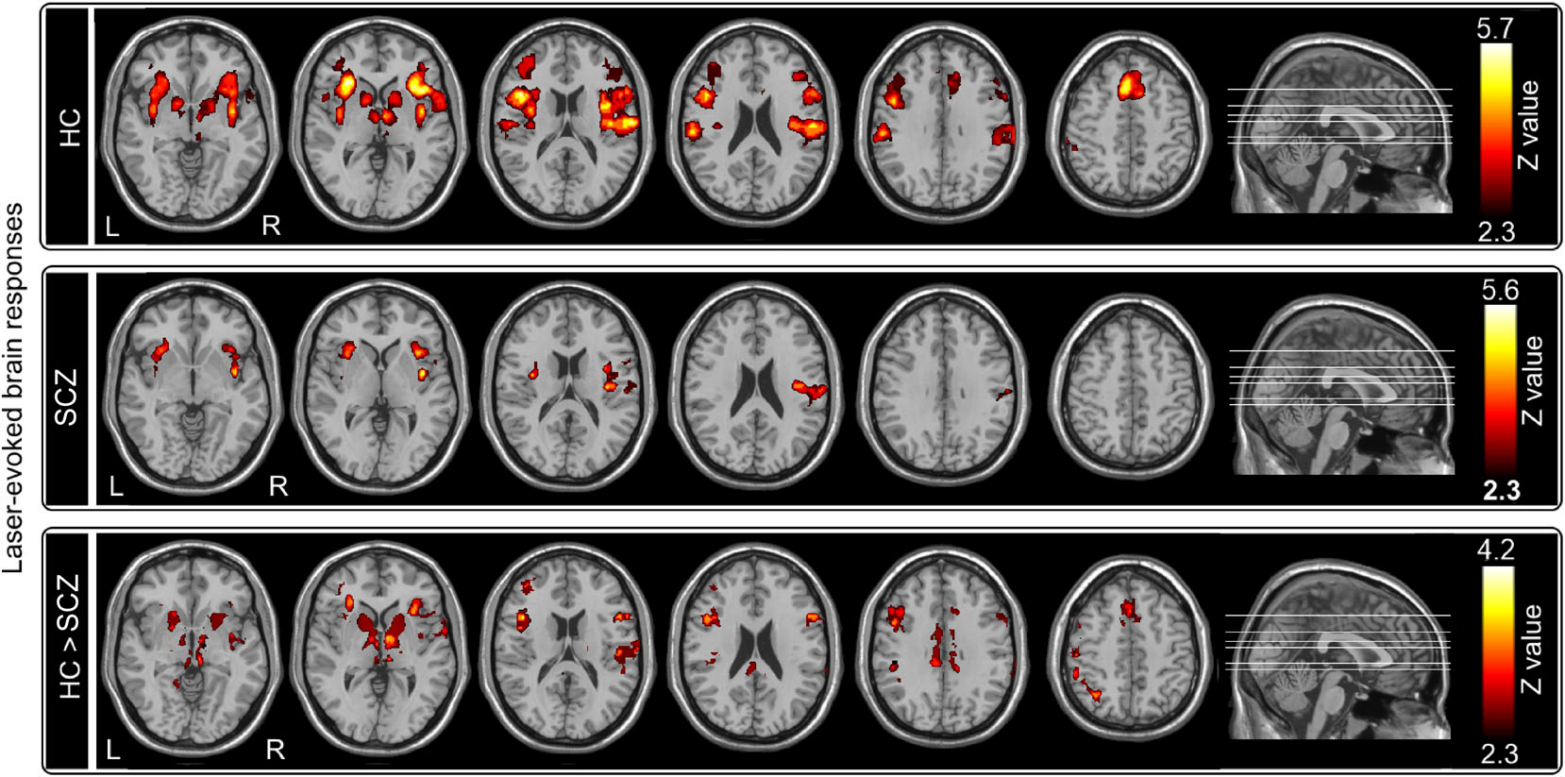
Event‐evoked BOLD responses to nociceptive stimuli. *Top panel*: For HC, nociceptive stimuli elicited significant activations in the PAG, thalamus, S1, S2, insula, and dACC. *Middle panel*: For SCZ, nociceptive stimuli elicited significant activations in the right S2 and bilateral insula. *Bottom panel*: Brain activations were significantly smaller in SCZ than HC in almost all brain regions associated with nociceptive information processing, including the PAG, thalamus, S2, insula, and dACC

Nociceptive stimuli elicited significant activations in a wide range of brain regions for HC, including the periaqueductal gray (PAG), thalamus, primary somatosensory cortex (S1), secondary somatosensory cortex (S2), insula, and dorsal anterior cingulate cortex (dACC) (*Z* > 2.3, *p <* .05 corrected; Figure 4, top panel). However, only the right S2 and bilateral insula were activated during the nociceptive stimuli for SCZ (*Z* > 2.3, *p* < .05 corrected; Figure 4, middle panel). Group‐level statistical results revealed that brain activations were significantly smaller in SCZ than HC in almost all brain regions associated with nociceptive information processing, including the PAG, thalamus, S2, insula, and dACC (*Z* > 2.3, *p* < .05 corrected; Figure 4, bottom panel). These results confirmed the systematic deficit of nociceptive information processing in SCZ.

### 3.4 Resting‐state fMRI functional connectivity

**Figure 5:**
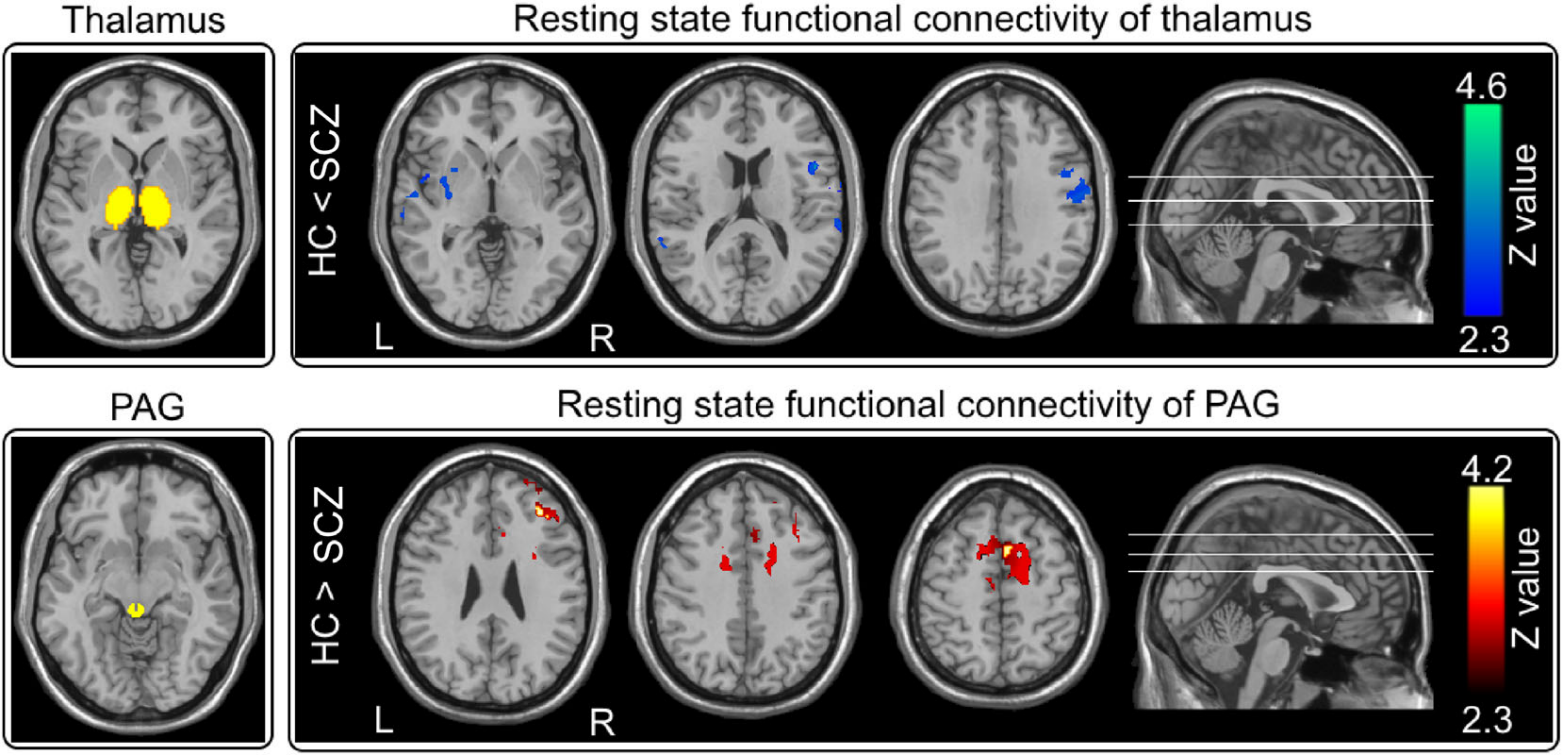
Resting‐state fMRI functional connectivity. *Top panel*: Thalamus showed weaker resting‐state functional connectivity with the right S1, right S2, left posterior insula in HC than in SCZ. *Bottom panel*: PAG had stronger resting‐state functional connectivity with the SMA, dACC, and DLPFC in HC than in SCZ

When thalamus was used as the ROI for the functional connectivity analysis of resting state fMRI data, we observed that thalamus exhibited weaker RSFC with the right S1, right S2, left posterior insula (PI) in HC than in SCZ (*Z* > 2.3, *p* < .05 corrected; Figure 5, top panel), suggesting an abnormal function of the ascending pain pathway at resting state in SCZ. In contrast, when PAG was used as the ROI for the same functional connectivity analysis, PAG showed stronger RSFC with the supplementary motor area (SMA), dACC, and dorsolateral prefrontal cortex (DLPFC) in HC than in SCZ (*Z* > 2.3, *p <* .05 corrected; Figure 5, bottom panel), indicating a possible degenerated function of the descending pain modulation pathway in SCZ.

### 3.5 Correlation results

When assessing the relationship between behavioral measures and laser‐evoked EEG responses, significant correlations were observed between ratings of perceived intensity and N1 amplitude (*r* = −0.548, *p* < .001), N2 amplitude (*r* = −.468, *p* = .002), as well as P2 amplitude (*r* = 0.483, *p* = .001; Table S2). When assessing the relationship between behavioral measures and prestimulus EEG oscillations, significant correlation was observed between ratings of perceived intensity and prestimulus lower alpha oscillations (*r* = −0.314, *p* = .043; Table S3).

When assessing the relationship between behavioral measures and laser‐evoked BOLD responses, significant correlations were observed between ratings of perceived intensity and BOLD responses to nociceptive stimuli in bilateral insula (*r* = 0.454, *p* = .007), as well as S2 (*r* = 0.339, *p* = .050; Table S4). When assessing the relationship between behavioral measures and resting‐state fMRI measures, significant correlation was observed between ratings of perceived intensity and RSFC of thalamus with right S2 (*r* = −0.396, *p* = .020; Table S3).

When assessing the relationship between EEG and fMRI measures at resting state, significant correlations were observed (1) between prestimulus lower alpha oscillations (6–7 Hz) and RSFC of thalamus with S1 (*r* = 0.533, *p* = .001) and S2 (*r* = 0.455, *p* = .007), and (2) between prestimulus higher alpha oscillations (9–10 Hz) and RSFC of thalamus with S1 (*r* = 0.539, *p* = .001; Table S3).

## 4 DISCUSSION

In the present study, combining with psychophysics, EEG, and fMRI techniques, we comprehensively investigated the difference of sensory processing across modalities between SCZ and HC. We obtained two main findings. First, SCZ patients were insensitive to nociceptive stimuli compared to HC, as revealed by three lines of evidence: (1) lower pain ratings (Table 2; Figure 2, top left panel), (2) lower amplitudes of all laser‐evoked EEG responses with longer latencies in the time domain (Table 2; Figures 2 and 3, top panels), and (3) lower laser‐evoked BOLD responses in almost all brain regions within the “pain matrix” (Figure 4, bottom panel). This finding is consistent with previous studies (de la Fuente‐Sandoval et al., 2010; de la Fuente‐Sandoval et al., 2012; Linnman et al., 2013), and could be related to the dysfunction of both ascending and descending pain modulation pathways in SCZ patients: thalamus exhibited stronger functional connectivity with the right S1, right S2, and left posterior insula, and PAG showed weaker functional connectivity with the SMA, dACC, and DLPFC in SCZ than HC (Figure 5). Second, even the perceived intensity ratings and the late part of neural responses (i.e., P2 wave) to non‐nociceptive somatosensory and auditory stimuli were not significantly different between SCZ and HC, N2 amplitude was significantly smaller in SCZ than HC (Table 2; Figures 2 and 3). This finding, which showed the dysfunction of sensory information processing across modalities, could be associated with the abnormality of the recurrent neuronal activity within the thalamocortical system in SCZ patients: compared with HC, the magnitudes of prestimulus alpha oscillations at both occipital and central electrodes were significantly larger in SCZ (Table S1; Figure S2, right panel; Figure S3).

### 4.1 Pain insensitivity in SCZ patients

Our observation that pain insensitivity in SCZ is supported by several previous studies (de la Fuente‐Sandoval et al., 2010; de la Fuente‐Sandoval et al., 2012; Linnman et al., 2013; Minichino et al., 2016). For instance, Minichino et al. (2016) demonstrated that compared to HC, SCZ patients had higher pain thresholds, and lower N1/N2/P2 amplitudes in laser‐evoked potentials. Additionally, de la Fuente‐Sandoval et al. (2010)) observed that drug‐naïve SCZ patients had a higher pain tolerance and a reduced activation in brain regions related to affective‐cognitive aspects of pain processing (insula and cingulate cortex) to thermal painful stimuli than HC. This pain insensitivity in SCZ patients was normally explained by a supra‐spinal mechanism involving bottom‐up and/or top‐down modulations in previous studies (de la Fuente‐Sandoval et al., 2010; Levesque et al., 2012; Potvin et al., 2008). For example, Levesque et al. (2012) observed that SCZ patients had a decreased sensitivity to prolonged pain, which was not accompanied by any difference in the nociceptive flexion reflex response. To achieve better understandings of the supra‐spinal mechanism, we performed seed‐based RSFC analyses for thalamus and PAG, which are key nodes in the ascending and descending pain modulation pathways respectively. We found that thalamus exhibited stronger RSFC with the right S1, right S2, left posterior insula in SCZ than in HC (Figure 5, top panel), which was consistent with numerous previous studies that highlighted the potential of the abnormal thalamocortical functional connectivity as a promising neurobiological marker to SCZ (Ferrarelli & Tononi, 2011; Giraldo‐Chica, Rogers, Damon, Landman, & Woodward, 2018; Welsh, Chen, & Taylor, 2010; Woodward, Karbasforoushan, & Heckers, 2012). Since thalamus is a key node responsible for transmissions of sensory signals in the ascending pain modulation pathway (Andersen & Dafny, 1983), the enhanced thalamocortical functional connectivity could indicate that the ascending pain pathway was hyper‐activated during rest in SCZ possibly due to sensory information overload. Therefore, the thalamocortical network dysfunction could provide an alternative explanation of pain insensitivity in SCZ. It is reasonable to speculate that overloaded irrelevant internal information (e.g., hallucination) in the thalamocortical network interrupt the transmission of nociceptive inputs, thus leading to the reduced pain experience. This surmise could be indirectly (at least partly) supported by a recent study that was focused on the role of excitatory and inhibitory systems in the pain modulation in SCZ patients applied a temporal summation paradigm before and after the activation of diffuse noxious inhibitory control (DNIC) system via a cold‐pressor test (Potvin et al., 2008). A lack of temporal summation in SCZ was observed in this study, which suggested a lack of central pain sensitization in patients, as temporal summation of pain is thought to reflect the progressive enhancement of C‐fiber (involving nociceptive information transmission) evoked responses in the central nervous system (Hu et al., 2014; Iannetti et al., 2003).

Additionally, we observed that PAG had weaker RSFC with the SMA, dACC, and DLPFC in SCZ than in HC (Figure 5, bottom panel), indicating a possible pain inhibition mechanism of the descending pain modulation pathway in SCZ contributed to their pain insensitivity. Extensive evidence has highlighted the importance of this descending pathway in modulating pain experience through inhibitory/excitatory mechanisms (De Felice et al., 2011; Tracey, 2017). As a crucial nucleus in the descending pathway, the enhanced RSFC of PAG to key brain regions of pain (e.g., the S1, thalamus and ACC) is responsible to central sensitization in chronic pain patients (Iannetti et al., 2005; Segerdahl et al., 2018; Zambreanu, Wise, Brooks, Iannetti, & Tracey, 2005), whose pain sensitivity is increased. Conversely, SCZ patients, whose pain sensitivity was decreased, showed attenuated RSFC of PAG to similar brain regions. Our observation was also supported by Potvin's finding, which DNIC significantly reduced pain perception in both SCZ and HC, but such reduction was more evident in HC than in SCZ (at the end of curve [80–120 s] in figure 1 in Potvin et al. (2008). Since DNIC involves an endogenous modulation mechanism triggered by nociceptive stimuli (Potvin et al., 2008), pain insensitivity in SCZ could not be due to the enhanced functioning of endogenous inhibitory systems. On the contrary, as the pain sensitivity is diminished in SCZ, there is no need for descending inhibitory system to modulate pain. As a longitudinal consequence, the function of the descending pain modulation pathway in SCZ could be degenerated according to the theory of use and disuse.

### 4.2 Abnormalities across sensory modalities in SCZ patients

In addition to pain insensitivity, dysfunction of sensory information processing across modalities was also observed in SCZ patients: the early part of brain responses (i.e., N2 amplitude) to non‐nociceptive somatosensory and auditory stimuli was significantly smaller in SCZ than HC (Table 2; Figures 2 and 3). Similar to LEP responses, vertex potentials elicited by intense stimuli belonging to different sensory modalities (Mouraux & Iannetti, 2009) largely reflect saliency‐related neural processes possibly related to the detection of relevant changes in the sensory environment (Downar, Crawley, Mikulis, & Davis, 2002). Considering that N2 wave is mainly generated from the insula that is an interoceptive integration brain structure playing a crucial role in the salience network, as it conveys multisensory information about internal body state and external surrounding environment (Craig, 2009), previous studies suggested that dysfunction of sensory information processing across modalities in SCZ patients could represent an epiphenomenon of salience network dysfunctions (Alustiza et al., 2018; Liddle et al., 2016; Minichino et al., 2016; Palaniyappan & Liddle, 2012; Potvin et al., 2008; Smucny, Wylie, Kronberg, Legget, & Tregellas, 2017). The salience network is involved in detecting and filtering salient stimuli and functions to segregate the most prominent information among internal and external stimuli in order to guide behavior (Legrain, Iannetti, Plaghki, & Mouraux, 2011; Mouraux, Diukova, Lee, Wise, & Iannetti, 2011). In accordance with this notion, the salience network dysfunction in SCZ patients would result in reduced ability to distinguish self‐initiated neural activity from neural activity evoked by external stimuli, which contributes to the dysfunction of sensory information processing across modalities and some psychotic symptoms, for example, hallucination (Palaniyappan & Liddle, 2012). Notably, we are aware that the majority of LEP responses are nonspecific to pain. However, they can still provide important information related to the state of the afferent nociceptive system, and be potentially useful to better understand the neural mechanisms of pain modulation through well‐designed control or longitudinal studies in clinical settings (Mouraux & Iannetti, 2018). For example, LEPs can help document the deficit of the nociceptive system (e.g., lesions in the spinothalamic tract), and thus are recommended as a diagnostic tool to distinguish patients with hyperalgesia or neuropathic pain from healthy populations (Treede, Lorenz, & Baumgartner, 2003).

Importantly, we observed that the magnitudes of prestimulus alpha oscillations were significantly larger in SCZ than HC (Table S1; Figure S2, right panel; Figure S3), which could also be associated with the dysfunction of sensory information processing across modalities. Noted that such brain oscillations reflect the discharging capacity of action potentials in thalamocortical relay neurons from tonic to burst modes depended on the neuronal membrane potentials (Llinas & Jahnsen, 1982). These state‐dependent oscillatory activities could characterize wakefulness/sleep, perceptual, and cognitive states (Buzsaki, Logothetis, & Singer, 2013; Freeman, 2006; Llinas, Urbano, Leznik, Ramirez, & van Marle, 2005) in an evolutionarily preserved way. Thus, our observation that the increased magnitudes of prestimulus alpha oscillations suggested an abnormal mental state at baseline in SCZ, even though such state‐dependent alpha oscillations could also be modulated by other factors, for example, level of vigilance, conscious awareness, and endogenous shifts of spatial attention (Linkenkaer‐Hansen, Nikulin, Palva, Ilmoniemi, & Palva, 2004; Mathewson, Gratton, Fabiani, Beck, & Ro, 2009; May et al., 2012). In fact, emerging evidence that altered brain oscillations in neurological diseases (e.g., Parkinson's disease and chronic pain) (Llinas, Ribary, Jeanmonod, Kronberg, & Mitra, 1999; Walton, Dubois, & Llinas, 2010) and neuropsychiatry disorders (e.g., depression and SCZ) (Schulman et al., 2011; Vanneste, Song, & De Ridder, 2018) has given rise to a postulated model known as thalamocortical dysrhythmia (TCD) (Llinas et al., 1999; Llinas et al., 2005; Vanneste et al., 2018). The major point behind the TCD model is that the generation of these intrinsic abnormal low‐frequency oscillations in the thalamocortical network could interrupt the original state‐dependent information flow between thalamus and cerebral cortex (Vanneste et al., 2018). In other words, these low‐frequency brain activities could serve as a trigger for the dysfunction of thalamocortical system, in which intrinsic neuronal properties form the substrate of illness‐related pathophysiology (Behrendt, 2006; Schulman et al., 2011). Specifically, the persistent thalamic neuronal hyperpolarization induced by the activation of low‐threshold (Cav3, T‐type) Ca^++^ channel and followed by the low‐frequency resonant recurrent interaction between thalamic and cortical neurons disrupted the normal function of thalamocortical circuit (Llinas, Ribary, Contreras, & Pedroarena, 1998). Notably, such hyperpolarization could occur by blocking the N‐methyl‐d‐aspartic acid receptors (NMDAr) in reticular thalamus and lead to the generation of low‐frequency brain oscillations, which is consistent with the major theories about the neuropathology of SCZ, including NMDAr hypofunction (Lindsley et al., 2006; Singh & Singh, 2011; Snyder & Gao, 2013), dopamine hyperfunction (Howes & Kapur, 2009; Lodge & Grace, 2011), and GABAergic neuronal inhibition (Gonzalez‐Burgos & Lewis, 2008; Gordon, 2010). Based on the above understandings of the intrinsic oscillatory properties of thalamic neurons and the biochemical effects of neurotransmitter system on the thalamocortical circuit, our results that the magnitude of prestimulus alpha oscillations was significantly larger in SCZ than HC suggested a different explanation: the abnormal sensory information processing across modalities in SCZ is related to the abnormal recurrent neuronal activity evidenced by the thalamocortical dysrhythmia.

### 4.3 Limitations and future directions

There are several limitations of our study. First, as all patients were receiving antipsychotic medication, we cannot exclude drug effects on pain sensitivity. Previous studies provided evidence showing that pain insensitivity in SCZ was independent of antipsychotic effects: (1) pain insensitivity in SCZ patients was reported before the introduction of antipsychotics (Hall & Stride, 1954; Marchand et al., 1959); (2) pain sensitivity was similarly reduced in both antipsychotic‐free and medicated patients (Potvin et al., 2008; Stubbs, Vancampfort, et al., 2015); (3) diminished pain sensitivity was observed in first‐degree relatives of SCZ patients (Hooley & Delgado, 2001). Nevertheless, these studies are rare and the antipsychotic effects on sensory perception are frequently addressed by pharmacologist (Catalani et al., 2014; Schreiber, Getslev, Backer, Weizman, & Pick, 1999). Thus, further studies on unmedicated patients are needed. Second, the small number of subjects (particularly the potential impact of patients' heterogeneity due to small sample size) limited the reliability and external validity of our findings. To testify the reliability of our results, we calculated values of Cohen's *d* for all statistical tests and further performed a post hoc test on the effectiveness of sample size. We obtained a large effect size of the detected group difference (Appelbaum et al., 2018; Cohen, 1992), and confirmed the sufficiency of our sample size (*n* = 42) with a large statistical power in the EEG experiment (1 − *β* = .99, determined by a large effect size of $\eta_{p}^{2}$ = 0.1339 or 1 − *β* = .82, determined by a large effect size of Cohen's *d* = 0.8 at the significance level of 0.05). Moreover, the fMRI results verify the findings of EEG experiment, thus increasing the reliability of our findings from different aspects. Admittedly, our findings still need to be replicated in a large and independent sample. Third, it is still unclear whether pain sensitivity varies with the development of the disease (i.e., acute, remission, chronic). To address this issue, our findings ought to be tested in prodromal‐phase or first‐episode patients in a longitudinal study, especially considering the tremendous differences of pain sensitivity across individuals (Hu & Iannetti, 2019). Forth, we did not detect the relationship between clinical symptoms (illness severity, positive or negative symptom, and cognitive impairments, etc.) and the dysfunction of pain processing due to the small sample size. Future studies could investigate the possible effects of these factors on pain perception in a multifactorial model, as the typical psychotic symptoms could be potential contributors to pain insensitivity in SCZ. Last but not the least, subsequent studies involving comparison of pain sensitivity between patients with SCZ versus other psychosis (e.g., major depressive disorder, bipolar disorder) are needed in the future to establish the specificity of this phenomenon in psychosis spectrum. After all the above‐mentioned issues have been clarified, our findings could be of great significance as neural index coded pain insensitivity could be used as a promising and intriguing trait marker for the diagnosis of SCZ in the future (Minichino et al., 2016).

## 5 CONCLUSIONS

Beyond a general dysfunction of cortical sensory information processing across modalities, the pain insensitivity in SCZ also relied on a specific deficit of ascending and descending pathways modulating nociceptive information processing. Our findings provide insights into the neural mechanisms of pain insensitivity in SCZ and highlight a need for systematic assessments of their pain‐related diseases.

## AUTHOR DECLARATION

All authors have seen and approved the final version of the manuscript being submitted. The article is an original work and has not received prior publication or been under consideration for publication elsewhere.

## CONFLICT OF INTEREST STATEMENT

All authors declared no competing interests.

## Supporting information


**Data S1** Supporting Information.

## Data Availability

The data that support the findings of this study are available from the corresponding author upon reasonable request. The data are not publicly available due to privacy or ethical restrictions.
