# Supplementary material for: A modality‐specific dysfunction of pain processing in schizophrenia
Source: Hum Brain Mapp. 2019 Dec 23;41(7):1738–53. doi: 10.1002/hbm.24906 (PMC7267942; doi:10.1002/hbm.24906)
Supplement: Supplementary file 1 — Data S1 Supporting Information. [file HBM-41-1738-s001.docx]

**Supplementary Materials for**

**“A modality-specific dysfunction of pain processing in schizophrenia”**

**Methods S1:** Time-frequency analysis

**Methods S2:** Prestimulus EEG oscillations

**Results S1:** Event-evoked EEG responses to different sensory stimuli in time-frequency domain

**Results S2:** Prestimulus EEG oscillations

**Table S1:** Comparisons of time-frequency features and prestimulus EEG oscillations between SCZ and HC (experiment 1)

**Table S2:** Correlations between subjective ratings of perceived pain and laser-evoked EEG responses (experiment 1)

**Table S3:** Correlations between subjective ratings of perceived pain and prestimulus EEG oscillations (experiment 1), as well as resting-state functional connectivity (experiment 2)

**Table S4:** Correlations between subjective ratings of perceived pain and laser-evoked BOLD responses (experiment 2)

**Figure S1:** Group-level event-related EEG responses to different sensory stimuli in the time-frequency domain

**Figure S2:** Comparisons of psychophysics and electrophysiological features between SCZ and HC

**Figure S3:** Group-level spectral power of prestimulus EEG oscillations

**Methods S1: Time-frequency analysis**

Time-frequency distributions (TFDs) of EEG epochs were obtained using a windowed Fourier transform (WFT) with a fixed 300-ms Hanning window. For each epoch, the WFT algorithm yielded a complex time-frequency estimate F(t, f) at each time-frequency point (t, f), which extended from -500 to 1000 ms (in steps of 1 ms) in the time domain, and from 1 to 30 Hz (in steps of 1 Hz) in the frequency domain. The resulting spectrogram, P(t, f)=|F(t, f)|^2^, represents the signal power as a joint distribution function at each time-frequency point. The spectrogram were baseline-corrected with a reference interval ranging from -400 to -100 ms at each frequency using the subtraction approach (i.e., subtracting the average power of the prestimulus interval from the power at each poststimulus time point) (Hu *et al.*, 2014). The reference interval was chosen to avoid the adverse inﬂuence of spectral estimates biased by windowing poststimulus activity and padding values. Previous studies demonstrated that sensory stimuli (nociceptive, non-nociceptive somatosensory, and auditory stimuli) can elicit large phase-locked (event related potentials, ‘ERP’) and non-phase-locked (event related desynchronization at alpha frequencies, ‘α-ERD’) responses (Pfurtscheller and Lopes da Silva, 1999; Mouraux *et al.*, 2003; Iannetti *et al.*, 2008; Hu *et al.*, 2014). Based on previous findings(Iannetti *et al.*, 2008; Peng *et al.*, 2012; Hu *et al.*, 2013), two regions of interest (ROIs) were defined to extract the magnitude of time-frequency responses within the baseline-corrected TFDs for each sensory modality (ROI1 for ‘ERP’: 100-400 ms, 1-10 Hz; ROI2 for ‘α-ERD’: 400-1000 ms, 8-12 Hz). Magnitudes of these time-frequency responses for each ROI were measured by computing the mean value of all time-frequency points within the given ROI for each subject and sensory modality. Group-level scalp topographies of the magnitude of each time-frequency response (i.e., ‘ERP’ and ‘α-ERD’) were computed by spline interpolation. Since ‘ERP’ and ‘α-ERD’ were maximally distributed at central (i.e., Cz) and occipital (i.e., PO3, PO4, PO5, PO6, PO7, and PO8) regions regardless of sensory modality (Peng *et al.*, 2012). The measured magnitudes at each ROI were compared using a two-way mixed design analysis of variance (ANOVA), with ‘modality’ as the within-subject factor (three levels: nociceptive, non-nociceptive somatosensory, and auditory) and ‘group’ as the between-subject factor (two levels: SCZ and HC). When the main effects or interactions reach statistical significance, post-hoc pairwise comparisons with Bonferroni correction were performed.

**Methods S2: Prestimulus EEG oscillations**

To assess the difference of prestimulus mental states between groups, we extracted prestimulus EEG signals from a time window (-1000 to 0 ms) relative to the stimulus onset, regardless of sensory modalities. For each subject and electrode, prestimulus EEG signals were transformed to the frequency domain using a discrete Fourier transform, yielding an EEG spectrum ranging from 1 to 30 Hz. Single-subject EEG spectra were averaged across subjects in each group, to obtain group-level prestimulus EEG spectra. To compare the group difference of prestimulus EEG spectra, we performed point-by-point independent-sample t-tests (i.e., each frequency point) for each electrode, with a false discovery rate (FDR) procedure (Benjamini *et al.*, 2001; Durka *et al.*, 2004). The results were then confirmed using region of interest (ROI) based analyses, where alpha oscillations (i.e., 6-7 Hz at occipital electrodes and 8-10 Hz at central electrodes) (Peng *et al.*, 2014; Tang *et al.*, 2015) were extracted and compared between groups using the independent-sample t-test.

**Results S1: Event-evoked EEG responses to different sensory stimuli in time-frequency domain**

Group-level time-frequency distributions, together with the scalp topographies of the ‘ERP’ and ‘α-ERD’ responses elicited by nociceptive stimuli are shown in the top panel of Figure S1A. Consistent with previous studies (Mouraux *et al.*, 2003; Schulz *et al.*, 2011; Peng *et al.*, 2012; Hu *et al.*, 2013), nociceptive stimuli elicited a large phase-locked response (‘ERP’: 100-400 ms, 1-10 Hz, maximal at central midline electrodes) and a clear non-phase-locked response (‘α-ERD’: 400-1000 ms, 8-12 Hz, maximal at parietal-occipital electrodes, bilaterally). Group-level time-frequency distributions, together with the scalp topographies of the ‘ERP’ and ‘α-ERD’ responses elicited by non-nociceptive somatosensory stimuli are shown in Figure S1B. Group-level time-frequency distributions, together with the scalp topographies of the ‘ERP’ and ‘α-ERD’ responses elicited by auditory stimuli are shown in Figure S1C.

For the magnitude of ‘ERP’, significant main effects of ‘group’ (F_(1,40)_=6.579, P=0.014, η_p_^2^=0.141) and ‘modality’ (F_(2,40)_=14.032, P<0.001, η_p_^2^=0.260), as well as significant interaction between ‘group’ and ‘modality’ (F_(2,40)_=5.288, P=0.010, η_p_^2^=0.117) were observed (Table S1; Figures S1 and S2). Post-hoc pairwise comparisons showed that the magnitude of ‘ERP’ elicited by nociceptive stimuli in SCZ was significantly smaller than that in HC (P=0.005), while no significant between-group differences were observed in the magnitudes of ‘ERP’ elicited by non-nociceptive somatosensory (P=0.486) and auditory (P=1.000) stimuli(Table S1; Figure S2, left panel).

For the magnitude of ‘α-ERD’, no significant main effect of ‘group’ (F_(1,40)_=0.042, P=0.839, η_p_^2^=0.001) and interaction between ‘group’ and ‘modality’ (F_(2,40)_=1.479, P=0.233, η_p_^2^=0.036) were observed. However, a significant main effect of ‘modality’ was observed (F_(2,40)_=4.036, P=0.021, η_p_^2^=0.092). Post-hoc pairwise comparisons showed that the magnitude of ‘α-ERD’ elicited by nociceptive stimuli were significantly larger than that by auditory stimuli (P=0.030; Table S1; Figure S1; Figure S2, middle panel).

**Results S2: Prestimulus EEG oscillations**

Group-level spectral power of prestimulus EEG oscillations, together with scalp topographies of oscillatory power at alpha frequencies (6 Hz, 7 Hz, 9 Hz, and 10 Hz), are showed in Figure S3. The spectral power at lower alpha frequencies (6-7 Hz) was maximal at fronto-central and occipital electrodes for both groups, and that at higher alpha frequencies (9-10 Hz) was maximal at occipital electrodes. When the signal was measured at occipital electrodes, mean magnitudes of spectral power at lower alpha frequencies were significantly larger in SCZ than HC (t(27)=-2.6, P=0.015, Cohen's d=-0.8) (Table S1; Figure S2, right panel; Figure S3, top panel). When the signal was measured at central electrodes, mean magnitudes of spectral power at higher alpha frequencies were also significantly larger in SCZ than HC (t(40)=-3.1, P=0.004, Cohen's d=-1.0) (Table S1; Figure S2, right panel; Figure S3, bottom panel).

**Table S1. Comparisons of time-frequency features and prestimulus EEG oscillations between SCZ and HC (experiment 1).**

| EEG features | Variables | SCZ (n=21) | HC (n=21) |
| --- | --- | --- | --- |
|  |  |  |  |
| LEP responses | ERP magnitude (μV/Hz) | 2.0±2.5 | 11.1±10.9 |
|  | α-ERD magnitude (μV/Hz) | 0.1±0.6 | -0.2±0.5 |
| SEP responses | ERP magnitude (μV/Hz) | 10.2±6.0 | 15.6±12.6 |
|  | α-ERD magnitude (μV/Hz) | -0.1±0.9 | -0.2±0.4 |
| AEP responses | ERP magnitude (μV/Hz) | 8.3±5.7 | 9.5±5.4 |
|  | α-ERD magnitude (μV/Hz) | -0.8±2.0 | -0.4±0.7 |
| Prestimulus EEG oscillations | Lower α power (μV/Hz) | 2.3±1.8 | 1.2±0.7 |
|  | Higher α power (μV/Hz) | 1.6±0.6 | 1.1±0.5 |

Note: Data are expressed in mean ± SD.

**Table S2. Correlations between subjective ratings of perceived pain and laser-evoked EEG responses (experiment 1).**

|  | N1 amplitude | N2 amplitude | P2 amplitude | ERP magnitude | α-ERD magnitude |
| --- | --- | --- | --- | --- | --- |
| Subjective rating | **-0.548^***^** | **-0.468^**^** | **0.483^***^** | **0.506^***^** | -0.078 |
| N1 amplitude |  | **0.768^***^** | **-0.655^***^** | **-0.697^***^** | -0.004 |
| N2 amplitude |  |  | **-0.734^***^** | **-0.866^**^** | 0.137 |
| P2 amplitude |  |  |  | **0.917^***^** | -0.010 |
| ERP magnitude |  |  |  |  | -0.024 |

Note: *, significant level at P=0.05 (2-tailed); **, significant level at P=0.01 (2-tailed); ***, significant level at P=0.001 (2-tailed).

**Table S3. Correlations between subjective ratings of perceived pain and prestimulus EEG oscillations (experiment 1), as well as resting-state functional connectivity (experiment 2).**

|  | PAG_SMA | PAG_ACC | PAG_DLPFC | Thalamus_S1 | Thalamus_S2 | Thalamus_PI |  | Lower α oscillations | Higher α oscillations |
| --- | --- | --- | --- | --- | --- | --- | --- | --- | --- |
| Subjective ratings | -0.133 | -0.228 | -0.002 | -0.278 | **-0.396^*^** | 0.055 |  | **-0.314^*^** | -0.270 |
| PAG_SMA |  | **0.723^***^** | **0.517^***^** | **-0.426^**^** | -0.235 | -0.289 |  | -0.157 | -0.261 |
| PAG_ACC |  |  | **0.518^***^** | **-0.334^*^** | -0.252 | -0.271 |  | 0.039 | 0.044 |
| PAG_DLPFC |  |  |  | -0.226 | 0.103 | **-0.469^**^** |  | -0.170 | -0.023 |
| Thalamus_S1 |  |  |  |  | **0.458^**^** | **0.321^*^** |  | **0.533^***^** | **0.539^***^** |
| Thalamus_S2 |  |  |  |  |  | 0.170 |  | **0.455^**^** | 0.320 |
| Thalamus_PI |  |  |  |  |  |  |  | 0.245 | 0.188 |
| Lower α oscillations |  |  |  |  |  |  |  |  | **0.766^***^** |

Note: *, significant level at P=0.05 (2-tailed); **, significant level at P=0.01 (2-tailed); ***, significant level at P=0.001 (2-tailed); PAG, periaqueductal gray matter; SMA, supplementary motor area; ACC, anterior cingulate cortex; DLPFC, dorsolateral prefrontal cortex; S1, primary somatosensory cortex; S2, secondary somatosensory cortex; PI, posterior insula; PAG_SMA, resting-state functional connectivity (RSFC) between PAG and SMA; PAG_ACC, RSFC between PAG and ACC; PAG_DLPFC, RSFC between PAG and DLPFC; Thalamus_S1, RSFC between Thalamus and S1; Thalamus _S2, RSFC between Thalamus and S2; Thalamus _PI, RSFC between Thalamus and PI.

**Table S4. Correlations between subjective ratings of perceived pain and laser-evoked BOLD responses (experiment 2).**

|  | Insula | Thalamus | PAG | S2 | ACC |
| --- | --- | --- | --- | --- | --- |
| Subjective ratings | **0.454^**^** | 0.211 | 0.306 | **0.339^*^** | 0.280 |
| Insula |  | **0.871^***^** | **0.613^***^** | **0.899^***^** | **0.898^***^** |
| Thalamus |  |  | **0.637^***^** | **0.909^***^** | **0.922^***^** |
| PAG |  |  |  | **0.658^***^** | **0.564^***^** |
| S2 |  |  |  |  | **0.909^***^** |

Note: *, significant level at P=0.05 (2-tailed); **, significant level at P=0.01 (2-tailed); ***, significant level at P=0.001 (2-tailed); PAG, periaqueductal gray matter; S2, secondary somatosensory cortex; ACC, anterior cingulate cortex.

**
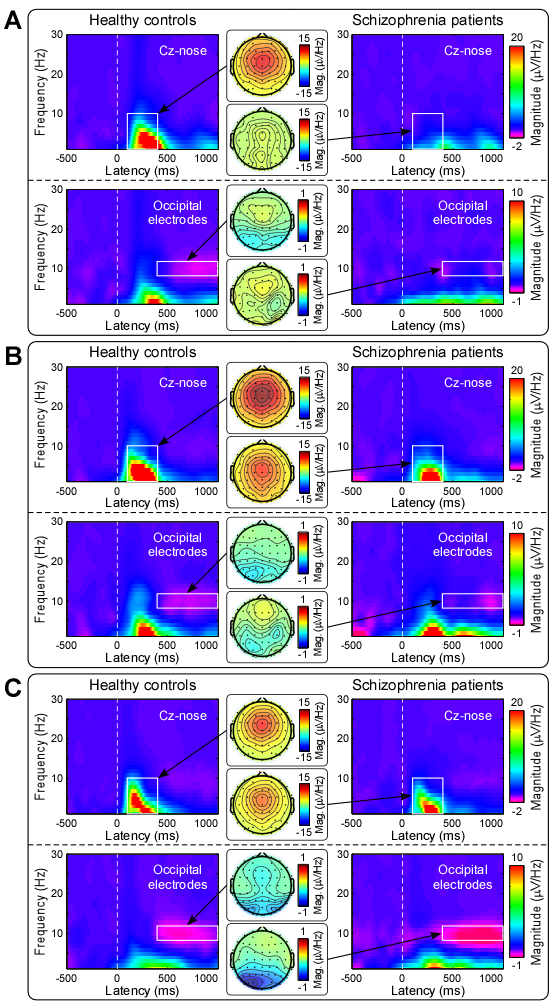
**

**Figure S1. Group-level event-related EEG responses to different sensory stimuli in the time-frequency domain.**

*A*: Group-level time-frequency distributions and scalp topographies of ‘ERP’ and ‘α-ERD’ responses to nociceptive stimuli. Data from SCZ and HC are displayed in right and left parts respectively. The color scale represents the increase or decrease of the oscillatory magnitude, relative to a prestimulus interval (-400 to -100 ms). The displayed time-frequency distributions contain both phase-locked (‘ERP’: 100-400 ms, 1-10 Hz) and non-phase-locked brain responses (‘α-ERD’: 400-1000 ms, 8-12 Hz), highlighted by the white rectangles. ‘ERP’ and ‘α-ERD’ magnitudes were maximal at central and parietal-occipital electrodes respectively.

*B*: Group-level time-frequency distributions and scalp topographies of ‘ERP’ and ‘α-ERD’ responses to non-nociceptive somatosensory stimuli.

*C*: Group-level time-frequency distributions and scalp topographies of ‘ERP’ and ‘α-ERD’ responses to auditory stimuli.

**
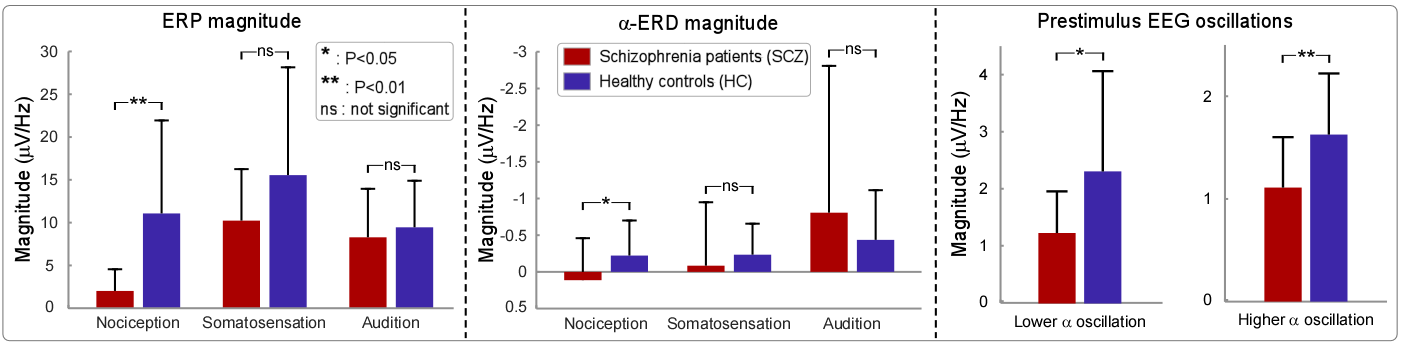
**

**Figure S2. Comparisons of psychophysics and electrophysiological features between SCZ and HC.**

*Left and middle panels*: Event-related EEG responses to different sensory stimuli in the time-frequency domain. Mean magnitude of ‘ERP’ elicited by nociceptive stimuli was significantly smaller in SCZ (red bar) than in HC (blue bar). Mean magnitude of ‘α-ERD’ elicited by nociceptive stimuli was significantly smaller than that elicited by auditory stimuli.

*Right panel*: Prestimulus EEG oscillations. Mean magnitudes of spectral power at both lower alpha (measured at occipital electrodes) and higher alpha (measured at central electrodes) frequencies were significantly larger in SCZ (red bar) than HC (blue bar) (*: P<0.05; **: P<0.01; ns: P>0.05).

**
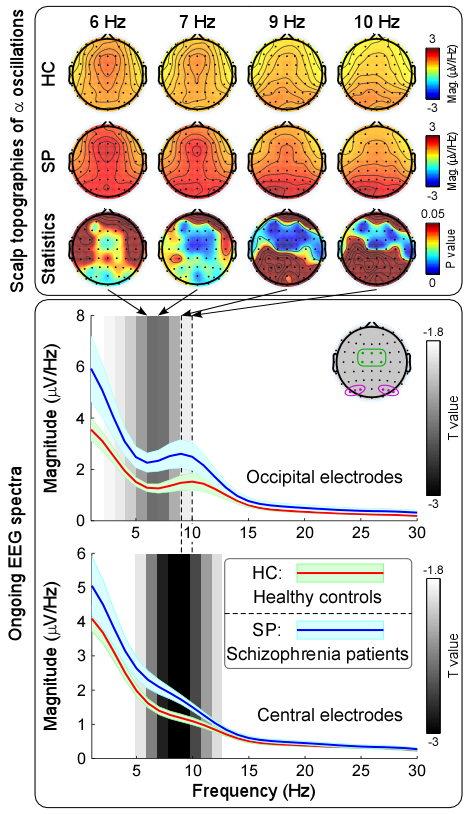
**

**Figure S3. Group-level spectral power of prestimulus EEG oscillations.**

*Top panel*: Scalp topographies of oscillatory power at alpha frequencies (6 Hz, 7 Hz, 9 Hz, and 10 Hz). For both SCZ and HC groups, the spectral power at lower alpha frequencies (6-7 Hz) was maximal at central and occipital electrodes, and that at higher alpha frequencies (9-10 Hz) was maximal at occipital electrodes. Significant differences of the spectral power between SCZ and HC were observed at occipital electrodes for lower alpha frequency, and at central electrodes for higher alpha frequency, respectively.

*Bottom panel*: Group-level spectral power of prestimulus EEG oscillations at occipital and central electrodes. Data from SCZ and HC are displayed in blue and red respectively. Frequency intervals with significant difference between the two groups are marked in grey (T values).

**References**

Benjamini Y, Drai D, Elmer G, Kafkafi N, Golani I (2001): Controlling the false discovery rate in behavior genetics research. Behav Brain Res 125(1-2): 279-84.

Durka PJ, Zygierewicz J, Klekowicz H, Ginter J, Blinowska KJ (2004): On the statistical significance of event-related EEG desynchronization and synchronization in the time-frequency plane. IEEE Trans Biomed Eng 51(7): 1167-75.

Hu L, Peng W, Valentini E, Zhang Z, Hu Y (2013): Functional features of nociceptive-induced suppression of alpha band electroencephalographic oscillations. J Pain 14(1): 89-99.

Hu L, Xiao P, Zhang ZG, Mouraux A, Iannetti GD (2014): Single-trial time-frequency analysis of electrocortical signals: baseline correction and beyond. Neuroimage 84: 876-87.

Iannetti GD, Hughes NP, Lee MC, Mouraux A (2008): Determinants of laser-evoked EEG responses: pain perception or stimulus saliency? J Neurophysiol 100(2): 815-28.

Mouraux A, Guerit JM, Plaghki L (2003): Non-phase locked electroencephalogram (EEG) responses to CO2 laser skin stimulations may reflect central interactions between A partial partial differential- and C-fibre afferent volleys. Clin Neurophysiol 114(4): 710-22.

Peng W, Hu L, Zhang Z, Hu Y (2012): Causality in the association between P300 and alpha event-related desynchronization. PLoS One 7(4): e34163.

Peng W, Hu L, Zhang Z, Hu Y (2014): Changes of spontaneous oscillatory activity to tonic heat pain. PLoS One 9(3): e91052.

Pfurtscheller G, Lopes da Silva FH (1999): Event-related EEG/MEG synchronization and desynchronization: basic principles. Clin Neurophysiol 110(11): 1842-57.

Schulz E, Tiemann L, Schuster T, Gross J, Ploner M (2011): Neurophysiological coding of traits and states in the perception of pain. Cereb Cortex 21(10): 2408-14.

Tang D, Hu L, Lei Y, Li H, Chen A (2015): Frontal and occipital-parietal alpha oscillations distinguish between stimulus conflict and response conflict. Front Hum Neurosci 9: 433.
